# Evaluate the Effects of Different Processing Methods on Red Dragon Fruit (*Hylocereus* species) Juice from the Perspective of Physicochemical Properties and Metabolic Profiles

**DOI:** 10.3390/foods14050793

**Published:** 2025-02-26

**Authors:** Shuai Chen, Yansong Li, Jiamu Kang, Congyan Su, Yuyi Liu, Yanfei Cheng, Zexin Wang, Shuxian Li, Congfa Li

**Affiliations:** 1 School of Food Science and Engineering, Hainan University, Haikou 570228, China; 17674726015@163.com (S.C.); 18834167777@163.com (Y.L.); kangjiamu@hainanu.edu.cn (J.K.); 21110832000007@hainanu.edu.cn (C.S.); 22220951350082@hainanu.edu.cn (Y.L.); chengyanfei1620@163.com (Y.C.); 18313570568@163.com (Z.W.); 22110832000009@hainanu.edu.cn (S.L.); 2 Key Laboratory of Food Nutrition and Functional Food of Hainan Province, Haikou 570228, China; 3 Key Laboratory of Tropical Agricultural Products Processing Technology of Haikou, Haikou 570228, China

**Keywords:** metabolomics, pasteurization, non-thermal processing, ultrasonic, cold plasma

## Abstract

Red dragon fruit juice (RDFJ) is a common fruit processing product on the market, and different processing methods can affect its quality. This study selected traditional thermal processing methods such as pasteurization and novel non-thermal processing methods like ultrasonication and cold plasma to treat red dragon fruit juice. The effects of different processing methods on the quality of red dragon fruit juice were comprehensively evaluated through physicochemical properties, in vitro activity, metabolomics and flavoromics. The results indicate that the cloud stability of cold plasma (CP) treatment increased by 16.64%, the total polyphenol content increased by 13.76%, and its antioxidant capacity was higher than that of other treatments. Cold plasma significantly increased the content of L-tyrosine and affected the amino acid metabolic pathways of RDFJ, which can contribute to the total phenolic content of RDFJ and antioxidant activity. CP treatment effectively preserved the aroma components of RDFJ and decreased the content of harmful furan volatile compounds. The results suggest that cold plasma is a promising innovative non-thermal processing technique in RDFJ.

## 1. Introduction

Pitaya (*Hylocereus polyrhizus*), commonly known as red dragon fruit, is an economically important crop primarily cultivated in tropical regions, particularly in Southern Mexico, as well as in Guangdong and Hainan provinces in China and other subtropical regions at similar latitudes [1]. Red dragon fruit is renowned for its high nutritional value, vibrant color, and sweet, refreshing taste. The juice extracted from red dragon fruit (RDF) is rich in bioactive compounds such as betacyanin, anthocyanins, flavonoids, vitamin, minerals, and polysaccharides, which are associated with numerous health benefits. Notably, betacyanin, polysaccharides, and polyphenols have antioxidant, anti-inflammatory, and anticancer properties [2]. As consumers increasingly opt for nutrient-dense foods, red dragon fruit offers an excellent source of essential nutrients, with its polyphenols promoting antioxidant activity and its polysaccharides and dietary fiber improving digestive health and alleviating constipation [3,4].

Despite its nutritional benefits, fresh RDF is highly perishable due to its high water content, which leads to spoilage during storage. Therefore, to extend its shelf life, the fruit is often processed into various products, with RDFJ being one of the most popular. Processing helps preserve the fruit’s nutritional and sensory qualities while mitigating issues like enzymatic browning and microbial contamination that could negatively impact juice quality [5]. During the production of RDFJ, it is essential to eliminate enzymes and microbial contaminants through thermal and non-thermal processing techniques.

The pasteurization method in thermal processing is widely used due to its simplicity and low cost [6]. However, this method can lead to a decline in juice quality, such as browning caused by heating and the degradation of heat-sensitive nutrients [7]. Ohmic heating and microwave heating are two new thermal processing techniques that have certain drawbacks. Due to the high equipment and time expenses, microwave heating is currently challenging to commercialize [8]. Non-thermal processing avoids the issue of nutrient loss in juice due to heating, gradually becoming a new choice for juice processing [9]. High-pressure processing is a popular non-thermal processing method. Applying high pressure to cell and protein structures renders pathogens in juice inactive [10]. Electric field processing is severely limited in its application due to its high cost, and there are also studies showing that the inactivation of enzymes by electric field processing is not good [11]. Ultrasonic treatment is a widely used non-thermal processing method that promotes the release of nutrients in fruit tissue by disrupting cell walls through the cavitation effects of sound waves. At the same time, enzymes and microorganisms are also inactivated due to the cavitation effect [12]. In recent years, a new non-thermal processing method called cold plasma has been applied to juice, showing great potential [13]. Cold atmospheric plasma, which consists of a complex mixture of UV photons, charged particles, and reactive species (e.g., reactive nitrogen species (RNS), reactive oxygen species (ROS), and reactive hydrogen species (RHS)), has shown considerable potential in food processing [14]. Recent studies have shown that cold plasma treatment can stabilize orange and tomato juice pigments and increase phenolic content [15]. However, there has been little investigation into using ultrasonic and cold plasma technologies for RDFJ. Most of the research on RDFJ has focused on fermentation, but there is missing research on RDFJ alone treatment to improve quality.

This study aims to investigate the effects of three different processing techniques—traditional pasteurization, ultrasonic processing, and cold plasma treatment—on the quality of RDFJ by examining the physicochemical properties, non-volatile compounds, and volatile compounds. It is hypothesized that non-thermal processing methods, such as ultrasonic processing and cold plasma treatment, will better preserve the nutritional and sensory qualities of red dragon fruit juice compared to traditional pasteurization. This research will provide a comprehensive understanding of how these processing methods impact the overall quality of RDFJ. The findings will offer valuable insights into the potential of non-thermal processing technologies for the production of high-quality RDFJ, laying a foundation for future innovations in fruit juice processing.

## 2. Materials and Methods

### 2.1. Materials and Reagents

In August 2024, a 50 kg fresh dragon fruit-picking event was conducted in Lingshui Li Autonomous County, Hainan Province, China. After picking, the fruits were transported to the lab in Hainan University and stored at 4 °C. Dragon fruits with uniform size and color and no mechanical damage were selected for the experiment. The fruits were peeled, cut into 1 cm × 1 cm pieces, and juiced by a Joyoung High Speed Wall Breaker (L18-P386, Joyoung Co., Ltd., Jinan Shandong China) for 2 min. The juice was filtered through 200-mesh gauze to obtain the red dragon fruit juice (RDFJ). All samples were frozen at −40 °C for later work, with 3 biological replicates for each treatment.

### 2.2. Sample Preparation

This study used three different treated RDFJ samples and a control group. The sample volume for all treatment groups was 100 mL. Untreated RDFJ samples were used as the control group (RAJ). Pasteurization was performed using the modified method of Jayachandran et al. [16] by placing RDFJ into a water bath (Shanghai Boxun Industrial Co., Ltd., Shanghai, China) preheated to 80 °C and treating it for 10 min to obtain sample PT. Ultrasonic cell disruptor (Q700, Qsonica, LLC, Newtown, USA) operated at 350 W power, working for 5 s, pausing for 6 s, with a total working time of 5 min at 20 kHz. The ultrasonic probe with a radius of 1.2 cm was located 2 cm below the juice surface, and the temperature was controlled at 25 °C using room temperature water. The obtained sample was labeled as US. The method was modified according to Dolas et al. [17]. Atmospheric plasma jet (APPJ) experimental equipment (PG-1000ZD, Nanjing Suman Plasma Engineering Research Institute Co., Ltd., Nanjing, China) used air as the excitation gas, with a flow rate of 0.6 L/min, voltage of 220 V, power of 300 W, and a treatment time of 30 s for the sample CP by modification of Dasan and Boyaci [15].

### 2.3. Determination of Cloud Value and Cloud Stability

Cloud value was measured using the method described by Illera et al. [18] with slight modifications. A 4 mL sample of red dragon fruit juice (RDFJ) was centrifuged at 760× *g* for 10 min in a thermostatic centrifuge at 4 °C. The supernatant was then collected, and the absorbance was measured at 660 nm.

Cloud stability was determined following the procedure outlined by Ertugay and Başlar [19]. A 4 mL sample of RDFJ was centrifuged at 4200× *g* for 15 min. The absorbance of the supernatant was measured at 625 nm. Cloud stability was calculated using Equation (1):

(1) $$\mathrm{CS}=\frac{A_{1}}{A_{2}}\times 100$$

where A_1_ and A_2_ represent the absorbances after and before centrifugation, respectively. In both measurements, absorbance was recorded using a DR6000 Hach Lange spectrophotometer, with distilled water serving as the blank.

### 2.4. Determination of Sedimentation Index and Browning Index

The sedimentation index was calculated with the method provided by Wu et al. [20] with some modifications. A 10 mL sample of red dragon fruit juice was transferred to a centrifuge tube and subjected to centrifugation at 1441× *g* for 15 min. The supernatant was then removed, and the tube was placed in an oven at 50 °C for 24 h. Afterward, the precipitate was weighed. The sedimentation index was calculated using Equation (2):

(2) $$\mathrm{SI}=\frac{m_{1}}{m_{2}}$$

where m_1_ is the mass of precipitate after centrifugation and m_2_ is the mass of RDFJ before centrifugation.

The browning index was measured using the method reported by Tiwari et al. [21] with some modifications. Amounts of 5 mL of RDFJ and 15 mL of 80% ethanol were mixed in a centrifuge tube, protected from light for 30 min, and centrifuged at 1441× *g* for 20 min at 4 °C. Absorbance values at 420 nm were determined, with 80% ethanol as a blank. The browning index was calculated according to Equation (3):

(3) $$\mathrm{BI}=\frac{V_{1}}{V_{2}}\times A$$

where V_1_ is the total volume of the extract, V_2_ is the sample volume and A is the absorbance value at 420 nm.

### 2.5. Determination of Total Phenol, Total Betacyanin and Reducing Sugar Content

Total phenolic content (TPC) was determined using the method described by Chen et al. [22] employing the Folin–Ciocalteu reagent. The TPC was calculated based on the standard curve and expressed as gallic acid equivalents (GAE) per gram (µg GAE/g). We took a 100 μL sample diluted 10 times after centrifugation of the supernatant, added 50 μL of Folin–Ciocalteu reagent, allowed a dark reaction for 5 min, added 300 μL of 20% Na_2_CO_3_ solution, allowed a dark reaction for 8 min, and then took 200 μL to determine the absorbance value at 765 nm. Reducing sugars were quantified using the 3,5-dinitrosalicylic acid (DNS) method: take 100 μL sample diluted 100 times after centrifugation of the supernatant, add 300 μL of DNS solution boiling water bath for 5 min, add 700 μL of distilled water to react for twenty minutes, and take 200 μL to determine the absorbance value at 540 nm. For the determination of total betacyanin content (TBC), the absorbance of the aqueous extract was measured at 538 nm using a spectrophotometer, and the concentration was calculated using Equation (4) [23].

(4) $$\mathrm{TBC}\left(\mathrm{mg}/\mathrm{mL}\right)=\left[\left(A\times \mathrm{MW}\times \mathrm{DF}\times 1000\right)/\left(E\times L\right)\right]$$

where A represents the absorbance at 538 nm, MW is the molecular weight of betacyanin (535), DF is the dilution factor, E is the molar extinction coefficient of betacyanin (60,000), and L is the path length (1 cm).

### 2.6. Determination of Polydispersity Index (PDI) and Particle Size

The concentration of the RDFJ was diluted 50 times and then analyzed using the Zetasizer Nano ZS90 (Malvern Instruments, Malvern, UK) to determine the average particle size and PDI. Dynamic light scattering technology was employed for this analysis, and three replicate experiments were conducted at an ambient temperature of 25 °C.

### 2.7. Determination of Organic Acid Content

The sample was centrifuged at 8000× *g* for 10 min to take the supernatant over a 0.22um organic filter membrane, and each sample was repeated three times.

For the quantitative determination of oxalic acid, L-tartaric acid, L-malic acid, and citric acid, an Agilent 1260 Infinity II high-performance liquid chromatography (HPLC) system (Santa Clara, CA, USA) was employed. This system was equipped with a 1260 variable-wavelength detector and an Agilent Zorbax SB-Aq C18 chromatographic column. The column had a length of 250 mm, an inner diameter of 4.6 mm, and a particle size of 5 μm.

The mobile phase was formulated by mixing a high-chloride solution (pH = 2.4) with methanol at a volume ratio of 98:2. During the entire analytical process, the mobile phase was isocratic and the column temperature was accurately controlled at 30 °C. A sample injection volume of 10 μL was chosen, and the flow rate of the liquid phase was set at 0.6 mL/min. The target chemical substances were detected and distinguished at a wavelength of 210 nm [24].

### 2.8. Determination of Antioxidant Activity and Enzyme Activity

The ABTS^+^ and DPPH free radical scavenging activities, as well as the Ferric reducing antioxidant power (FRAP), were assessed according to the manufacturer’s instructions utilizing kits purchased from Nanjing Jiancheng Co., Ltd. (Shanghai, China). The activities of polyphenol oxidase (PPO) and peroxidase (POD) enzymes were evaluated utilizing kits bought from Grace Biotechnology Co., Ltd. (Suzhou, China).

DPPH free radical scavenging activities were determined as follows: 50 μL of a 20-fold diluted sample was taken, mixed and reacted for 30 min, and the absorbance value was determined at 517 nm.

(5) $$\mathrm{DPPH}\;\mathrm{free}\;\mathrm{radical}\;\mathrm{scavenging}\;\mathrm{activities}\;\left(\%\right)=1\;-\;\frac{A_{1}-A_{2}}{A_{3}}$$

A1 = sample + DPPH, A2 = sample + water, A3 = water + DPPH. (All are absorbance values measured at 517 nm).

ABTS^+^ free radical scavenging activities were determined as follows: 50 μL of a 20-fold diluted sample was taken, mixed and reacted for 30 min, and the absorbance value was determined at 734 nm.

(6) $${\mathrm{ABTS}}^{+}\;\mathrm{free}\;\mathrm{radical}\;\mathrm{scavenging}\;\mathrm{activities}\;\left(\%\right)=1\;-\;\frac{A_{1}-A_{2}}{A_{3}}$$

A1 = sample + ABTS^+^, A2 = sample + water, A3 = water + ABTS^+^. (All are absorbance values measured at 734 nm).

Ferric reducing antioxidant power was determined as follows: 300 mmol/L CH_3_COONa solution, 10 mmol/L tripyridyltriazine solution, and 20 mmol/L Fecl_3_ solution were mixed in the ratio of 10:1:1 and stored at 37 °C. A total of 30 μL of 20-fold diluted samples was added to 900 μL of the mixture and reacted for 30 min away from light, and the absorbance value was determined at 593 nm. The measured value was subtracted from the absorbance value at 593 nm of the same volume of distilled water reacted with the mixture for 30 min and substituted into the standard curve. The antioxidant capacity of the samples was expressed in Trolox equivalents.

For the determination of PPO enzyme activity, we used the following method: Take 0.1 g of sample and add 1 mL of extraction solution. Homogenize after ice lustration, centrifuge at 4 °C, 12,974× *g* for 15 min, take 50 μL of sample, and add the reaction reagent at 25 °C. Determine the absorbance value A_1_ at 420 nm, and after 5 min, determine the absorbance value A_2_. Substitute the value of A_2_-A_1_ into the formula to calculate the PPO enzyme activity.

For the determination of POD enzyme activity, we used the following method: Take 0.1 g of sample and add 1 mL of extraction solution. Homogenize after ice lustration, centrifuge at 4 °C, 12,974× *g* for 15 min, take 10 μL of sample, and add the reaction reagent at 25 °C. Determine the absorbance value A_1_ at 470 nm, and after 1 min, determine the absorbance value A_2_. Substitute the value of A_2_-A_1_ into the formula to calculate the POD enzyme activity.

### 2.9. Liquid Chromatography–Ion Trap–Time of Flight–Mass Spectrometry (LC-IT-TOF-MS) Analysis

In this stage, we used the following method: First, pipette 100 μL of RDFJ into a clean centrifuge tube. Next, add 400 μL of a mixed extraction solvent, which is composed of acetonitrile and methanol in an equal volume ratio (1:1), to the tube. The resulting mixture is then subjected to ultrasonication at 5 °C and 40 kHz for 30 min. Once the sonication is completed, place the centrifuge tube in a −20 °C refrigerator for 30 min to facilitate protein precipitation.

Subsequently, centrifuge the mixture at 13,000× *g* and 4 °C for 15 min. Transfer the obtained supernatant to a new centrifuge tube and evaporate it under a high-purity nitrogen gas stream. After that, redissolve the residue with 100 μL of a reconstitution solution, which consists of a 1:1 mixture of acetonitrile and water, and conduct another sonication treatment at 5 °C and 40 kHz for 5 min.

Following the second ultrasonication, centrifuge the sample once more at 13,000× *g* and 4 °C for 15 min. Collect the supernatant and transfer it into a sample vial for subsequent analysis by LC-IT-TOF-MS (Shimadzu, Kyoto, Japan). Inject a 5 μL sample into the system and perform separation using an ACQUITY^®^ HSS T3 column (100 mm × 2.1 mm × 1.8 μm, sourced from Shanghai, China) for mass spectrometry-based detection.

The mobile phase is composed of two distinct solvents. Solvent A is a mixture of water and acetonitrile (95:5) containing 0.1% formic acid. Solvent B consists of acetonitrile and water (95:5 *v*/*v*) with 0.1% formic acid.

The solvent gradient is detailed as follows: during the initial period from 0 to 5.5 min, the proportion of solvent B gradually increases from 0% to 24.5%. Between 5.5 and 9 min, there is a change in the proportion of solvent B, which rises from 24.5% to 65%. In the time interval of 9 to 15.4 min, the proportion of solvent B ranges from 65% to 100%. Subsequently, from 15.4 to 16 min, it decreases from 100% to 51.5%. Finally, in the period from 16 to 20 min, it further drops from 51.5% to 0%.

The injection volume of the sample into the system is set at a constant value of 5 μL. The flow rate of the mobile phase is adjusted to 0.4 mL/min, and the temperature of the chromatographic column is precisely controlled at 40 °C. During the whole analytical process, all samples are maintained at a steady temperature of 4 °C.

Mass spectrometric data are acquired using an LC-IT-TOF-MS device (manufactured by Shimadzu, Japan), which operates in both positive and negative ion modes. The optimum parameters are as follows: the heater temperature is set at 425 °C, the capillary temperature at 325 °C, the sheath gas flow rate at 50 arbitrary units (arb), and the auxiliary gas flow at 13 arb. The ion-spray voltage floating is set to −3500 V in negative ion mode and +3500 V in positive ion mode.

The normalized collision energy for mass spectrometry varies within the range of 20–40–60 V. The full-scale mass spectrometry resolution is configured at 60,000, whereas the mass spectrometry resolution is documented as 7500. Data collection is performed in Data Dependent Acquisition mode, and the detection encompasses a mass-to-charge ratio (*m*/*z*) range from 80 to 1050 [25].

### 2.10. Headspace Microextraction–Solid-Phase Microextraction Combination with Gas Chromatography Mass Spectrometry (HS-SPME-GC-MS) Analysis

A total of 1 mL of RDFJ was added to a 20 mL headspace vial that had been previously filled with 5 μL of the internal standard, 4-methyl-2-pentanol at a concentration of 20 μg/mL. The extraction process was accomplished using a DVB/CAR/PDMS fiber (sized 50/30 μm), which was sourced from Supelco in Bellefonte, Pennsylvania, USA.

The analytical apparatus employed a combined approach of headspace microextraction and solid-phase microextraction. In particular, an HS-SPME-GC-MS system (model 6890N/5977B, produced by J&W Agilent, Santa Clara, USA) was used. This system was outfitted with a VF-MAX chromatographic column (30 m × 0.25 mm × 0.25 μm; supplied by J&W Agilent, Santa Clara, USA).

The temperature profile commenced with an initial isothermal period at 50 °C for 2 min. This was followed by a linear temperature ramp at a rate of 3.5 °C per min until the temperature reached 230 °C, which was then held for an additional 10 min. The electron impact ionization (EI) energy was fixed at 70 eV. Mass spectrometry data were obtained in full-scan mode over a mass-to-charge ratio (*m*/*z*) range spanning from 35 to 350.

Under consistent experimental conditions, n-alkanes in the range of C8–C40 were applied as external standards for the determination of retention indices. To identify volatile organic compounds, the mass spectra of the unknown substances were compared with the reference spectra from the NIST Library Version 17 L. This comparison was made in conjunction with their respective retention index (RI) values. Quantification was accomplished by comparing the peak areas of the analytes with that of the internal standard [26].

### 2.11. Statistical Analysis

Statistical analyses were carried out using GraphPad Prism 9.5 (San Diego, CA, USA). The data are presented as the mean ± standard deviation. One-way analysis of variance (ANOVA), followed by Duncan’s test, was utilized to assess statistical differences. Significance was defined as *p* < 0.05.

Partial least squares discriminant analysis and orthogonal projections to latent structures discriminant analysis (OPLS-DA) were performed with MetaboAnalyst 5.0 (https://www.metaboanalyst.ca/MetaboAnalyst/home.xhtml, accessed on 15 January 2025) and SIMCA software (version 14.1, Umetrics, Umeå, Sweden). The data were pre-processed by Par scaling prior to these multivariate statistical analyses.

## 3. Results

### 3.1. The Effects of Different Processing Methods on the Physicochemical Properties of RDFJ

**Figure 1:**
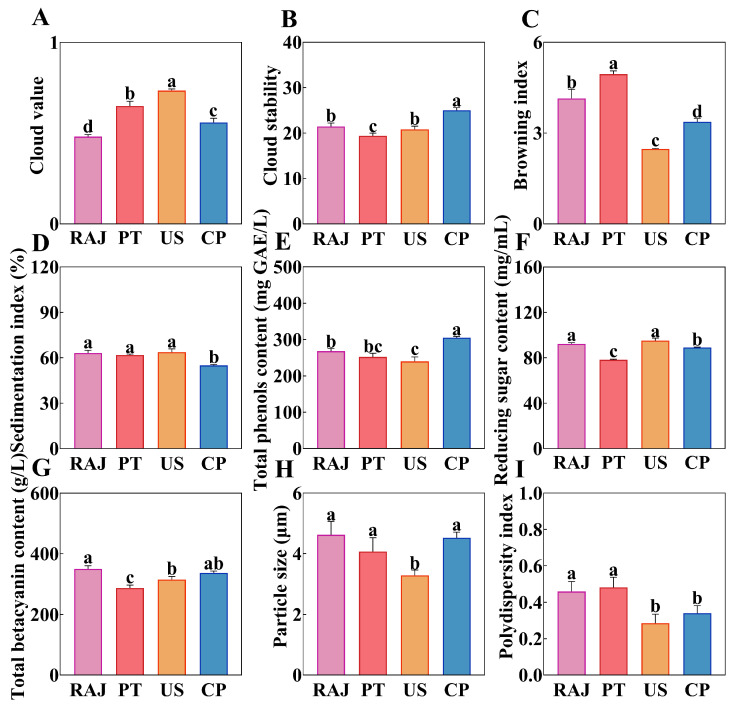
The impact of different treatments on the physicochemical properties of RDFJ is shown in the following parameters: (**A**) cloud value, (**B**) cloud stability, (**C**) browning index, (**D**) sedimentation index, (**E**) total phenol content, (**F**) reducing sugar content, (**G**) total betacyanin content, (**H**) particle size, and (**I**) polydispersity index. Different letters indicate statistical significance, determined by one-way ANOVA (*p* < 0.05). Data are presented as mean ± SD (n = 3). Note: RAJ, raw juice; PT, pasteurized juice; US, ultrasonic-treated juice; CP, cold plasma juice.

The cloud value and cloud stability are important parameters for evaluating RDFJ, as they reflect the stability of the juice system [27]. As shown in Figure 1A, the cloud value of RAJ was 0.48 ± 0.01, while by the cold plasma jet after treatment, the cloud value increased to 0.56 ± 0.02. The cloud value of the PT treatment increased to 0.65 ± 0.02, and the cloud value of the US treatment increased to 0.74 ± 0.01. When using ultrasonic and cold plasma treatments, solid particles and suspended matter in the juice may break up or reorganize due to their mechanism of action, resulting in changes in the turbidity of the juice. Ultrasonic treatment may increase the turbidity of the juice by breaking larger pulp particles into smaller particles, while cold plasma may maintain lower turbidity by reducing microbial and enzyme activity [28]. The results indicated that the CP treatment exhibited the highest cloud stability, consistent with the findings regarding cloud values (Figure 1B).

Different processing methods can affect the interactions among these volatile compounds within the juice, thereby impacting the overall stability of the juice system [29]. The browning index provides a direct reflection of the sensory characteristics of fruit juice (Figure 1C). The main causes of juice browning are enzymatic browning and the Maillard reaction [30]. US and CP treatment’s browning index was significantly lower than pasteurization. This indicates that both US and CP treatments effectively inhibited the juice’s enzymatic and non-enzymatic browning processes. As shown in Figure 1D, the sedimentation index of CP treatment was 54.91 ± 0.52%, which showed that CP treatment improved the stability of RDFJ. As shown in Figure 1E, different processing methods significantly impacted the content of reduced sugars in the fruit juice. Following PT treatment, the concentration of reducing sugars in the juice decreased markedly, which may be attributed to the Maillard reaction induced by heating [31]. The primary active components in the dragon fruit juice were betacyanin and polyphenols. As shown in Figure 1F,G, significant differences were observed in the content of these two volatile compounds across different groups. The total phenolic content of CP treatment was higher than other treatments, this phenomenon may be due to the cold plasma disrupting the cell wall, causing phenolic compounds to be released from within the cells [32]. The betacyanin content in PT treatment significantly decreased. Betacyanin is a type of pigment that easily reacts when heated. Notably, the betacyanin content in CP treatment decreased the least, indicating that the cold plasma has a minimal impact on betacyanin [33]. As shown in Figure 1H,I, both the particle size and PDI of the US treatment were at their lowest levels; however, its cloud value was at its highest. This may be due to the cavitation effect of ultrasonication, which reduces the particle size in the juice and disperses it more evenly. This finding was consistent with results observed in ultrasonic-treated apple juice [34].

From the perspective of physicochemical properties, CP treatment not only improved cloud stability and reduced browning index results but also performed better in increased total phenol content and reduced betacyanin loss. These results indicated that cold plasma was a promising method for treating RDFJ.

### 3.2. The Impact of Different Processing Methods on the Organic Acids, Enzyme Activity, and Antioxidant Activity of RDFJ

**Figure 2:**
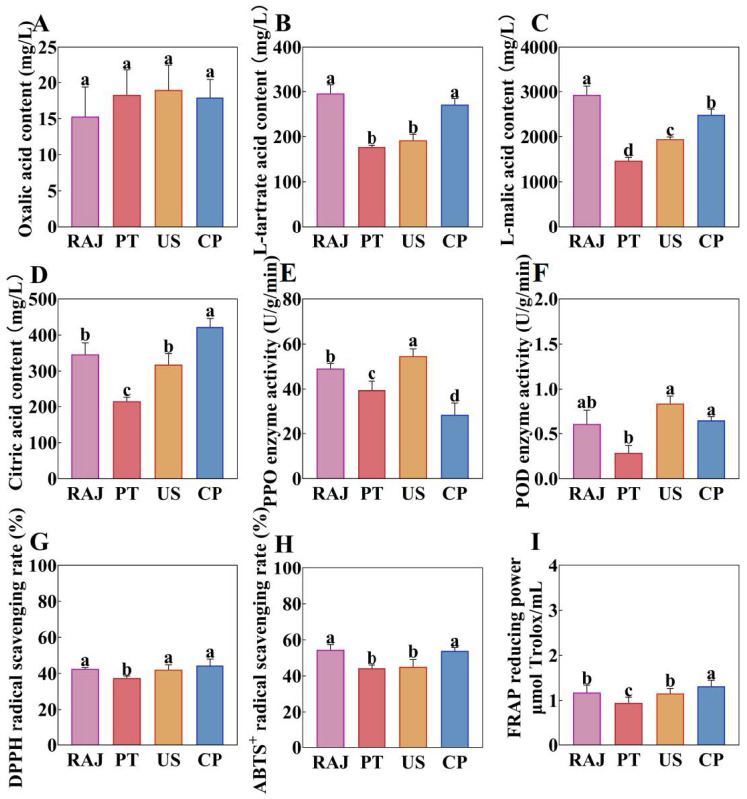
The impact of different treatments on the organic acids, enzyme activity, and antioxidant capacity of RDFJ. (**A**) Oxalic acid, (**B**) L-tartrate acid, (**C**) L-malic acid, (**D**) L-malic acid, (**E**) PPO enzyme activity, (**F**) POD enzyme activity, (**G**) DPPH radical scavenging rate, (**H**) ABTS^+^ radical scavenging rate, (**I**) FRAP reducing power. Statistical significance is indicated by different letters, with a one-way ANOVA (*p* < 0.05) used to determine significance. Data are presented as Mean ± SD (n = 3).

Organic acids are essential nutrients in fruit juices and play a crucial role in determining the sensory attributes of food [35]. The content of oxalic acid, L-tartaric acid, L-malic acid, and citric acid in RDFJ processed with different methods was determined using HPLC. Among these, L-malic acid was the predominant organic acid in RDFJ. Other studies have also reported L-malic acid as the main organic acid in RDFJ [36]. As shown in Figure 2A–D, the L-malic acid content was significantly higher than other organic acids. All three treatments resulted in varying degrees of reduction in L-malic acid content, with PT treatment showing the greatest decrease and CP treatment showing the least reduction. The oxalic acid content remained unchanged across the different treatments, while L-tartaric acid exhibited similar trends to L-malic acid. Notably, the citric acid content in the CP treatment was significantly higher than in the other treatments, likely due to cold plasma’s potential effect on the metabolic pathways of RDFJ, which may promote the conversion of citric acid [37]. As seen in Figure 2E,F, the activity of PPO and POD enzymes in CP-treated RDFJ was significantly reduced. This reduction in enzyme activity may be due to the damage caused by active nitrogen and oxygen species in the cold plasma jet, leading to enzyme inactivation. Enzyme inactivation can slow down enzyme-catalyzed browning, helping maintain the quality of RDFJ [38]. During cold plasma treatment of mangosteen juice, a significant decrease in PPO and POD enzyme activities was also observed, corroborating the reliability of the experimental results [39]. The decreased enzyme activity observed during PT treatment could be attributed to the increased temperature, which can change enzyme structures and even cause inactivation [40]. In Figure 2G–I, the antioxidant capacity of RDFJ treated by different methods shows noticeable variations. CP treatment exhibited the best antioxidant capacity, while PT treatment had the poorest. The antioxidant capacity of RDFJ is primarily related to the content of betacyanin and polyphenols [41]. In the CP treatment, the higher content of betacyanin and polyphenols compared to other treatments supports this finding. This is consistent with a study that found increased total polyphenol content after tomato juice was treated with cold plasma [42]. We needed to study the reasons for the changes in physicochemical properties further, so we determined the changes in metabolites.

### 3.3. The Impact of Different Processing Treatments on the Metabolic Profile of Dragon Fruit Juice

**Figure 3:**
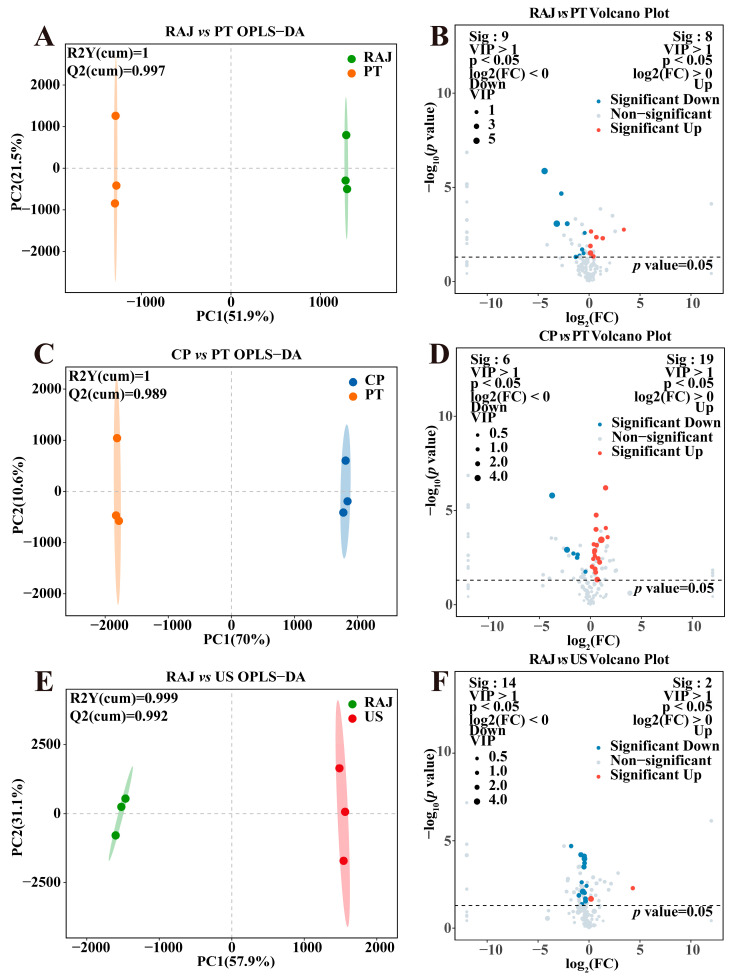
(**A**) RAJ vs. PT OPLS-DA score plot; (**B**) RAJ vs. PT volcano plot; (**C**) CP vs. PT OPLS-DA score plot; (**D**) CP vs. PT volcano plot; (**E**) RAJ vs. US OPLS-DA score plot; (**F**) RAJ vs. US volcano plot. The significance of dotted lines is a *p* value of 0.05.

**Figure 4:**
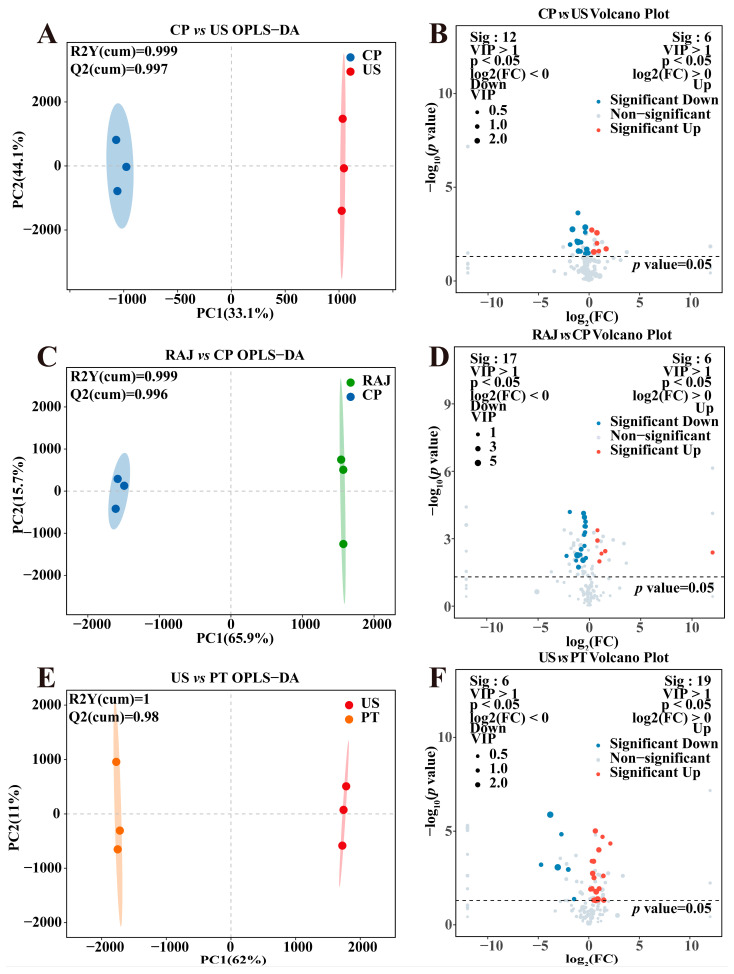
(**A**) CP vs. US OPLS-DA score plot; (**B**) CP vs. US volcano plot; (**C**) RAJ vs. CP OPLS-DA score plot; (**D**) RAJ vs. CP volcano plot; (**E**) US vs. PT OPLS-DA score plot; (**F**) US vs. PT volcano plot. The significance of dotted lines is a *p* value of 0.05.

We calculated the variable importance in projection (VIP) values to assess the significance of metabolites in relation to the variables. Differential metabolites were identified using the criterion of VIP > 1, *p* < 0.05, and FC > 1 or <1. We combined the metabolites identified from both positive and negative ion modes and utilized OPLS-DA models and volcano plots to examine significantly different metabolites across diverse processing methods. As shown in Figure 3A,B, the OPLS-DA plot has R^2^Y(cum) = 1 and Q^2^(cum) = 0.997, demonstrating that the model has good predictive performance. The results of the volcano plot indicated that in the comparison between RAJ treatment and PT treatment, 17 differential metabolites were identified, with 8 metabolites showing increased abundance and 9 metabolites showing decreased abundance. As seen in Figure 3C,D, the OPLS-DA plot has R^2^Y(cum) = 1 and Q^2^(cum) = 0.989, demonstrating that the model has good predictive performance. The results of the volcano plot indicate that in the comparison between CP treatment and PT treatment, 25 differential metabolites were identified, with 19 metabolites showing increased abundance and 6 metabolites showing decreased abundance. The OPLS-DA plot has R^2^Y(cum) = 0.999 and Q^2^(cum) = 0.992, demonstrating that the model has good predictive performance (Figure 3E,F). The results of the volcano plot indicate that in the comparison between RAJ treatment and US treatment, 16 differential metabolites were identified, with 2 metabolites showing increased abundance and 14 metabolites showing decreased abundance. The OPLS-DA plot has R^2^Y(cum) = 0.999 and Q^2^(cum) = 0.997, demonstrating that the model has good predictive performance (Figure 4A,B). The results of the volcano plot indicate that in the comparison between CP treatment and US treatment, 18 differential metabolites were identified, with 6 metabolites showing increased abundance and 12 metabolites showing decreased abundance. As shown in Figure 4C,D, the OPLS-DA plot has R^2^Y(cum) = 0.999 and Q^2^(cum) = 0.996, demonstrating that the model has good predictive performance. The results of the volcano plot indicate that in the comparison between RAJ treatment and CP treatment, 23 differential metabolites were identified, with 6 metabolites showing increased abundance and 17 metabolites showing decreased abundance. In Figure 4E,F, the OPLS-DA plot has R^2^Y(cum) = 1 and Q^2^(cum) = 0.98, demonstrating that the model has good predictive performance. In all six scatter plots, two distinctly separated groups are evident, with cumulative predictive scores (R^2^Ycum > 0.9) indicating a strong model fit and excellent predictive capability (Q^2^cum > 0.9) [43]. Based on the OPLS-DA model and the volcano plots, we have found that the RDFJs of the different treatments were entirely differentiated. Next, it is essential to analyze the specific differential metabolites between the different groups.

### 3.4. LC-IT-TOF-MS Investigated the Impact of Various Processing Techniques on Non-Volatile Components in RDFJ

**Figure 5:**
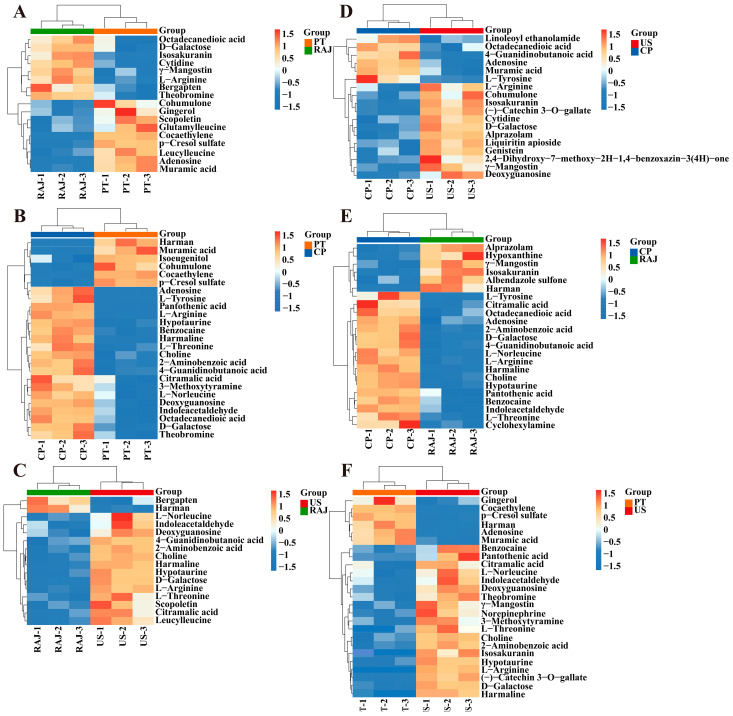
Clustering heatmap of differential metabolites under different treatments. (*p* < 0.05, VIP > 1). (**A**) PT vs. RAJ; (**B**) PT vs. CP; (**C**) US vs. RAJ; (**D**) US vs. CP; (**E**) CP vs. RAJ; (**F**) PT vs. US.

The color blocks of the cluster heatmap can intuitively reflect the changes in metabolite abundance. Six cluster heatmaps were drawn to analyze the differential metabolites of RDFJ under different processing treatments. As shown in Figure 5A, the abundance of bergapten, γ-Mangostin, theobromine, isosakuranin, octadecanedioic acid, L-Arginine and cytidine significantly decreased in the PT treatment. Bergapten is a natural coumarin compound, and Isosakuranin is a natural flavonoid compound. They demonstrate anti-inflammatory properties by obstructing the NF-κB pathway, hence diminishing the synthesis of inflammatory mediators such as IL-6, IL-1β, and TNF-α [44,45]. γ-Mangostin is a flavonoid compound that can inhibit the activity of cyclooxygenase (COX-2) and reduce the expression of inflammatory factors [46]. The decrease in the abundance of bergapten and γ-Mangostin will affect the antioxidant capacity of RDFJ, which is consistent with the experimental results showing the lowest DPPH radical scavenging rate and ABTS^+^ radical scavenging rate in the PT treatment. As shown in Figure 5B, the abundance of pantothenic acid, adenosine, L-Tyrosine and hypotaurine was increased. Pantothenic acid, also known as vitamin B5, can scavenge free radicals and has antioxidant properties [47]. Hypotaurine acts as an intermediate in taurine biosynthesis. It effectively scavenges superoxide anions (O_2_·^−^) within the body, playing a crucial role in shielding cells from oxidative injury. This scavenging function contributes to the improvement of the antioxidant capacity of RDFJ, highlighting its significance in maintaining cellular health and potentially enhancing the overall quality of RDFJ in terms of its antioxidant properties [48]. As shown in Figure 5C, in US treatment, the decrease in the abundance of bergapten and the increase in the abundance of D-Galactose can induce oxidative stress, increase inflammatory responses, and lead to a decrease in the antioxidant activity of RDFJ [49]. Amino acids are important nutrients that enhance the nutritional value of RDFJ [50]. As a result, the abundance of adenosine and L-Tyrosine was increased in the CP treatment (Figure 5D). Among, adenosine was a naturally occurring nucleotide that inhibits the release of pro-inflammatory cytokines by activating the A2A receptor [51]. As shown in Figure 5E, the CP treatment increased the abundance of L-tyrosine, L-Arginine, L-threonine, pantothenic acid, hypotaurine, and adenosine. Hypotaurine and adenosine provide RDFJ with antioxidant and anti-inflammatory capabilities. The important thing is that the free amino acids serve as precursors of aromatic compounds (alcohols, aldehydes, and ketoacids), and different processing methods and changes in amino acid composition could influence the aromatic profile of RDFJ. In addition, the abundance of (-)-Catechin 3-O-gallate and Isosakuranin increased in the US treatment (Figure 5F). Catechin 3-O-gallate and Isosakuranin both belong to flavonoid compounds, which have strong free radical scavenging abilities, enhancing the antioxidant capacity of RDFJ [52]. Next, we subjected these differential metabolites to pathway enrichment analysis, focusing on which metabolic pathways they specifically affect to alter RDFJ quality.

### 3.5. Analysis of Differential Metabolite Metabolic Pathways

**Figure 6:**
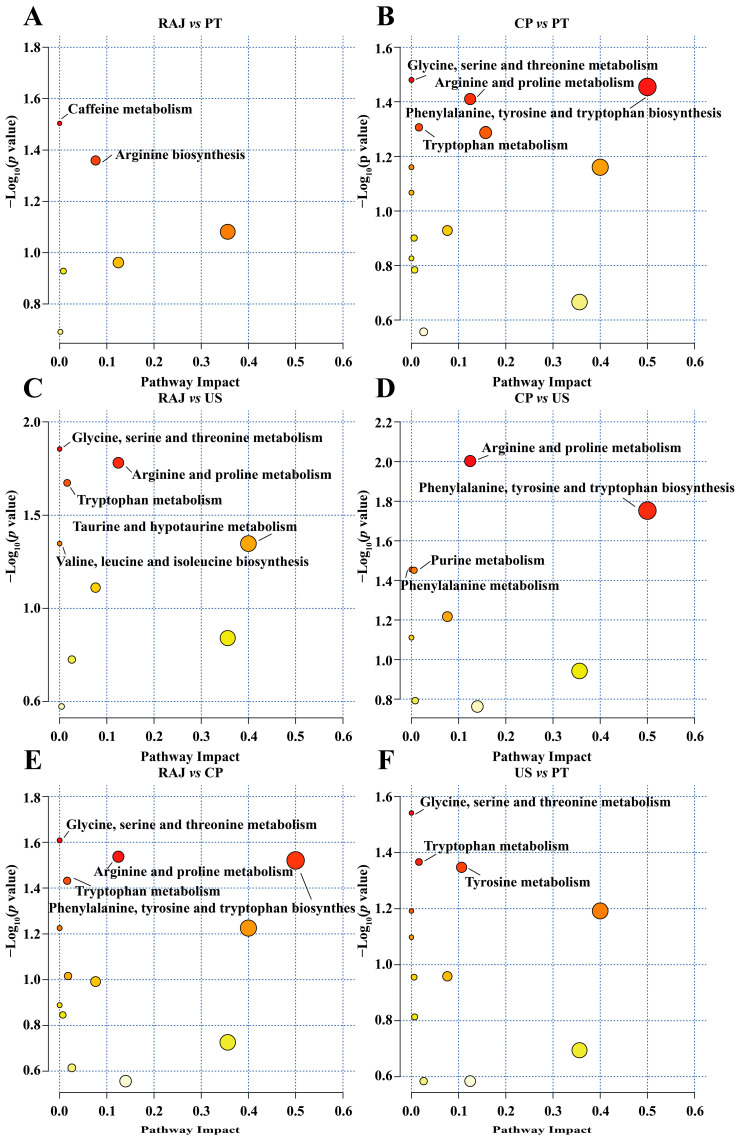
KEGG enrichment pathway analysis of differential metabolites. (*p* < 0.05). (**A**) RAJ vs. PT; (**B**) CP vs. PT; (**C**) RAJ vs. US; (**D**) CP vs. US; (**E**) RAJ vs. CP; (**F**) US vs. PT.

Analyzing differential metabolite metabolic pathways provides a comprehensive understanding of the changes in biological processes. Using the KEGG database, we annotated the pathways related to differential metabolites [53]. In comparing RAJ and PT treatments, significant enrichment was observed in the caffeine metabolism and arginine biosynthesis pathways (Figure 6A). When comparing CP and PT treatments, several metabolic pathways were enriched, including glycine, serine, and threonine metabolism; arginine and proline metabolism; phenylalanine, tyrosine, and tryptophan biosynthesis; and tryptophan metabolism (Figure 6B). When comparing RAJ and US treatments, glycine, serine, and threonine metabolism; arginine and proline metabolism; tryptophan metabolism; taurine and hypotaurine metabolism; and valine, leucine, and isoleucine biosynthesis were enriched (Figure 6C). Similarly, when comparing CP and US treatments, we found enrichment in the pathways of arginine and proline metabolism, phenylalanine, tyrosine, and tryptophan biosynthesis, purine metabolism, and phenylalanine metabolism (Figure 6D). In the comparison between RAJ and CP treatments, enrichment was observed in pathways like glycine, serine, and threonine metabolism; arginine and proline metabolism; phenylalanine, tyrosine, and tryptophan biosynthesis; and tryptophan metabolism (Figure 6E). Additionally, comparing US and PT treatments, the enriched metabolic pathways included glycine, serine, threonine metabolism, tryptophan metabolism, and tyrosine metabolism (Figure 6F). Notably, the pathways of phenylalanine, tyrosine, and tryptophan biosynthesis, as well as glycine, serine, and threonine metabolism, appeared in multiple pairwise comparisons, suggesting their potential role as key pathways responsible for the metabolic differences in RDFJ resulting from various processing methods.

Further analysis revealed that L-Tyrosine, an important functional metabolite linked to the phenylalanine, tyrosine, and tryptophan biosynthesis pathways, was found at significantly higher levels in CP-treated RDFJ than in other treatments. Amino acids are important factors affecting the flavor of fruit juices, and changes in amino acids during processing may lead to alterations in flavor, especially for the perception of sweetness, freshness, and mouthfeel of the juice [54]. Meanwhile, the phenylalanine biosynthesis pathway was significantly influenced, which plays a crucial role in synthesizing lignin, flavonoids, and phenolic compounds [55]. In contrast, pasteurization may lead to the degradation of certain amino acids, which may, in turn, affect the flavor [56]. Previous studies have demonstrated that cold plasma treatment can enhance the gene expression and activity of key enzymes in the phenylalanine pathway, promoting phenolic accumulation and boosting antioxidant activity [57]. These findings help explain why RDFJ treated with cold plasma exhibited the highest total phenolic content (TPC) and the strongest antioxidant activity.

### 3.6. HS-SPME-GC-MS Investigation of the Impact of Various Processing Techniques on Volatile Components in RDFJ

**Figure 7:**
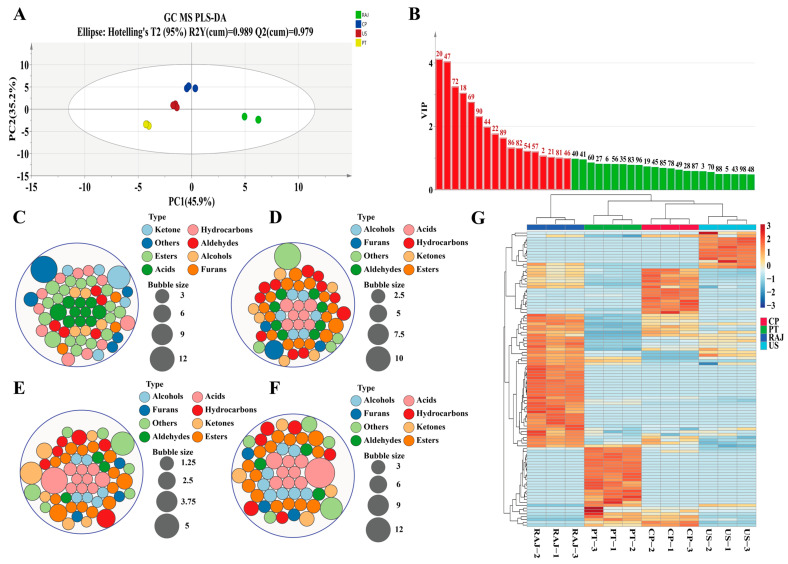
Flavoromics VOC multivariate statistical analysis. (**A**) PLS-DA score plot; (**B**) VIP values of VOCs (red represents 17 compounds with VIP > 1); (**C**) RAJ treatment VOC quantity classification bubble chart; (**D**) PT treatment VOC quantity classification bubble chart; (**E**) US treatment VOC quantity classification bubble chart; (**F**) CP treatment VOC quantity classification bubble chart; (**G**) clustering heatmap of 157 VOCs under different treatments. Note: 20, Dimethyl ether; 47, Acetic acid; 72, Acetoin; 18, Coumaran; 69, 4-Methyl-2-pentanone; 90, Ethyl caprylate; 44, Palmitoleic acid; 22, 1,2,4-TRIMETHYL BENZENE; 89, Ethyl palmitate; 86, Ethyl tetradecanoate; 82, 1,3-dimethylbutyl acetate; 54, (+/–)-13-methyl-tridecanolide; 57, Tridecane; 2, (2R,3R)-(–)-2,3-Butanediol; 21, 4-Ethyltoluene81, Ethyl laurate; 46, palmitic acid.

Volatile organic compounds (VOCs) are crucial in determining the overall sensory quality of RDFJ [58]. Using HS-SPME-GC-MS, 104 types of VOCs were detected in the RDFJ samples. The total concentration of VOCs showed varying degrees of reduction across different treatments. Specifically, the PT treatment led to a 43.14% reduction, the US treatment a 53.16% reduction, and the CP treatment a 27.73% reduction (Table S1). The most significant reductions were observed in esters, ketones, and acids. Pasteurization (PT) results in a greater release and conversion of volatile compounds in juice, while non-thermal treatments, such as CP and US, are more effective at preserving these compounds [59]. Other studies have also demonstrated that cold plasma retains substances better and reduces damage to flavor compounds [60]. We used the PLS-DA model to identify key differential VOCs, which highlighted metabolites with a VIP > 1 as important for distinguishing the treatments. As shown in Figure 7A, there were significant flavor differences among the various RDFJ treatments. Based on the VIP > 1 criterion, 17 differential VOCs were selected (Figure 7B). In the RAJ treatment (Figure 7C), 63 VOCs were detected, including 6 ketones, 18 esters, 10 acids, 11 hydrocarbons, 5 aldehydes, 5 alcohols, 3 furans, and 5 other compounds. In the PT treatment (Figure 7D), 62 VOCs were detected, with 6 ketones, 16 esters, 8 acids, 11 hydrocarbons, 8 aldehydes, 6 alcohols, 3 furans, and 4 other compounds. The US treatment (Figure 7E) showed 57 VOCs, including 6 ketones, 15 esters, 10 acids, 6 hydrocarbons, 3 aldehydes, 7 alcohols, 3 furans, and 7 other compounds. Finally, the CP treatment (Figure 7F) revealed 54 VOCs, with 4 ketones, 15 esters, 9 acids, 6 hydrocarbons, 3 aldehydes, 9 alcohols, 3 furans, and 5 other compounds.

Furan compounds, potentially carcinogenic and toxic, were detected in all four treatments. The formation of furan compounds is typically attributed to the Maillard reaction, where amino acids react with reducing sugars and lipid oxidation, including polyunsaturated fatty acids and ascorbic acid [61]. The content of furan compounds in PT treatment was the highest, far exceeding that of other treatments, while the content of furan compounds in CP treatment was the lowest. This proved that pasteurization increased the content of harmful volatile compounds in RDFJ, thereby reducing its quality. Aldehydes mainly originate from the oxidation of fatty acids and the lipoxygenase pathway in fruit juice. Three types of aldehydes were detected in both US and CP treatments, while eight were detected in PT treatment. Among these, the highest concentration in PT treatment was (E)-2-nominal, a volatile compound with a soapy taste that can induce an unpleasant feeling.2,4-dimethyl benzaldehyde is a volatile compound with a cherry aroma. Additionally, it possesses antioxidant properties and anti-tumor effects, which can extend juice’s shelf life. All treatments were detected to be 2,4-dimethyl benzaldehyde except for PT treatments.

Acids are essential for the juice’s characteristic sour taste, influencing flavor and mouthfeel and acting as antioxidants to protect juice nutrients from oxidation. The reduction in acid content was most significant in PT treatment, while CP treatment showed increased acid content. Palmitoleic acid and palmitic acid, both key differential metabolites with a VIP > 1, were present at higher concentrations in CP treatment and lower concentrations in PT treatment. Palmitoleic acid is a monounsaturated fatty acid that, when combined with other fatty acids, can lower cholesterol levels and reduce inflammation [62]. Palmitic acid, though not a strong antioxidant by itself, can work synergistically with other polyphenolic compounds to enhance the overall antioxidant capacity of RDFJ [63]. Hydrocarbons, including monoterpenes, sesquiterpenes, and aromatic hydrocarbons, contribute to the aroma and flavor of RDFJ. The content of hydrocarbons decreased significantly across all treatments. Regarding ketones, the CP treatment had the least reduction in their content, with acetoin, a key flavor compound with a creamy aroma, notably retained. In PT treatment, the acetoin content was 0.062 ± 0.01 μg/mL, while in CP treatment, it was 2.738 ± 1.475 μg/mL. This demonstrates that CP treatment better preserves acetoin, which helps maintain the quality of RDFJ. Esters are important for enhancing the aroma of RDFJ, and the content and variety of esters were relatively high across treatments. In the CP treatment, the levels of ethyl caprate, ethyl nominate, and ethyl acetate were all higher than in the PT treatment. With fruity, brandy, and cherry sweetness aromas, these esters significantly enriched the flavor profile of RDFJ.

Figure 7G shows significant changes in the volatile compounds were observed across different treatments. While many volatile compounds decreased, new ones, such as heptyl acetate and costol, appeared in the CP treatment. This suggests that CP treatment retained the aroma compounds better than PT and US treatments, indicating its advantage in preserving the quality and sensory attributes of RDFJ.

### 3.7. Analysis of the Correlation Between Key Differential Metabolites, Enzyme Activity, and Antioxidant Activity

An analysis was conducted on the correlation between differential metabolites with VIP > 1 screened by metabolomics and flavoromics and their antioxidant and enzyme activity.

**Figure 8:**
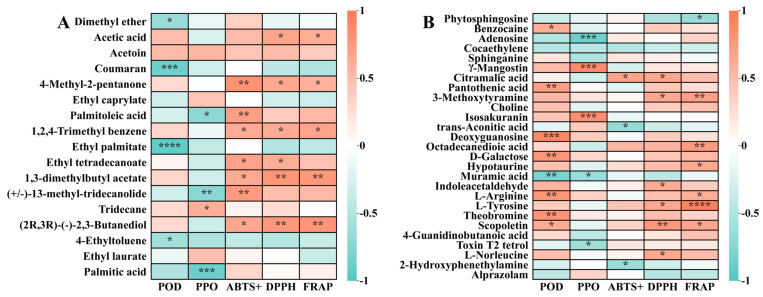
Correlation analysis of differential metabolites with POD enzyme activity, PPO enzyme activity, DPPH, ABTS^+^, and FRAP. DPPH, DPPH radical scavenging ability; ABTS^+^, ABTS^+^ radical scavenging ability; FRAP, Ferric reducing antioxidant power. (**A**) Correlation analysis of 17 metabolites detected by HS-SPME-GC-MS (VIP > 1). (**B**) Correlation analysis of 29 metabolites detected by LC-IT-TOF-MS (VIP > 1). * *p* < 0.05, ** *p* < 0.01, *** *p* < 0.001, **** *p* < 0.0001 indicate significant difference.

As shown in Figure 8A, orange represents a positive correlation, and green represents negative correlation. The analysis based on the Euclidean distance Spearman correlation algorithm indicates that 4-Ethyltoluene, dimethyl ether, and POD enzyme activity are significantly negatively correlated (*p* < 0.05), while Coumaran, ethyl palmitate, and PDO enzyme activity are extremely significantly negatively correlated (*p* < 0.001). Palmitoleic acid, (+/–)-13-methyl-tridecanolide, palmitic acid, and PPO enzyme activity are significantly negatively correlated (*p* < 0.05). Tridecane is significantly positively correlated with PPO enzyme activity (*p* < 0.05). 4-Methyl-2-pentanone, 1,2,4-Trimethyl benzene, 1,3-dimethylbutyl acetate, (2R,3R)(–)-2,3-Butanediol is significantly positively correlated with DPPH and ABTS^+^ radical scavenging ability and FRAP (*p* < 0.05). Acetic acid is significantly positively correlated with DPPH radical scavenging ability and FRAP (*p* < 0.05), palmitoleic acid is significantly positively correlated with ABTS^+^, ethyl tetradecanoate is significantly positively correlated with ABTS^+^ and DPPH (*p* < 0.05), and (+/−)-13-methyl-tridecanolide is significantly positively correlated with ABTS^+^ (*p* < 0.05).

As shown in Figure 8B, the citramalic acid and ABTS^+^ radical scavenging abilities were significantly positively correlated (*p* < 0.05), and trans-Aconitic acid, 2-Hydroxyphenethylamine and ABTS^+^ radical scavenging abilities were significantly negatively correlated (*p* < 0.05). The citramalic acid, 3-Methoxytyramine, indoleacetaldehyde, L-Tyrosine, scopoletin, L-Norleucine and DPPH radical scavenging abilities were significantly positively correlated (*p* < 0.05). The indoleacetaldehyde, L-Tyrosine, scopoletin, L-Norleucine and DPPH radical scavenging abilities were significantly positively correlated (*p* < 0.05). There was a significant negative correlation between phytosphingosine and FRAP (*p* < 0.05). 3-Methoxytyramine, octadecanedioic acid, hypotaurine, L-Arginine, and scopoletin were significantly positively correlated with FRAP (*p* < 0.05). L-tyrosine was positively correlated with FRAP (*p* < 0.0001).

The results of this study indicate that cold plasma treatment leads to a remarkable increase in the content of functional volatile compounds within RDFJ. This enhancement effectively improves the functional properties and bioactivity of RDFJ. Nevertheless, the underlying mechanisms governing the synthesis and decomposition processes of numerous metabolites remain poorly understood. Therefore, further in-depth research is essential to clarify these mechanisms and provide a more comprehensive understanding of the impact of cold plasma treatment on RDFJ.

## 4. Conclusions

This study utilized LC-IT-TOF-MS and HS-SPME-GC-MS techniques to comprehensively investigate the effects of traditional thermal processing methods (pasteurization) and novel non-thermal processing methods (ultrasonication and cold plasma) on both non-volatile and volatile components of red dragon fruit juice (RDFJ). The results demonstrated significant changes in the metabolite profiles of RDFJ depending on the processing method applied. The LC-IT-TOF-MS analysis revealed a significant increase in the content of key differential metabolites such as adenosine, theobromine, scopoletin, and L-tyrosine (VIP > 1) following cold plasma treatment, which improves the antioxidant activity of RDFJ. Furthermore, cold plasma treatment enhanced the accumulation of phenolic compounds in RDFJ by modulating the phenylpropanoid biosynthetic pathway, which in turn improved the antioxidant capacity of the juice. In addition, the HS-SPME-GC-MS results showed that key volatile compounds, including phenethyl alcohol, acetoin, 2,4-dimethyl benzaldehyde, and palmitoleic acid (VIP > 1), were present in higher concentrations in the cold plasma-treated RDFJ compared to other treatments. Meanwhile, the content of harmful volatile compounds was decreased. Phenethyl alcohol and acetoin, both with creamy and floral aromas, contributed to enhancing the flavor profile of RDFJ. On the other hand, 2,4-dimethyl benzaldehyde and palmitoleic acid are known for their antioxidant and anti-inflammatory properties. They further enhanced the antioxidant activity of RDFJ.

In conclusion, the ability of cold plasma to significantly improve the quality of RDFJ by modulating its metabolite profile provides valuable insights into the potential applications of cold plasma in the food industry, particularly for enhancing the nutritional and sensory attributes of fruit juices. Subsequent studies should pay further attention to the detailed discussion of the potential long-term effects of cold plasma treatment and treatment parameters in order to obtain better experimental results. At the same time, more efficient cold plasma equipment should be developed to reduce costs and avoid limitations in its use.

## Data Availability

The original contributions presented in this study are included in the article/Supplementary Material. Further inquiries can be directed to the corresponding author.

## Supplementary Materials

The following supporting information can be downloaded at: https://www.mdpi.com/article/10.3390/foods14050793/s1, Table S1: Concentrations of 157 VOCs; Table S2: pH and TS of different treatments.

## Abbreviations

HS-SPME-GC-MS: headspace microextraction–solid-phase microextraction combination with gas chromatography mass spectrometry; LC-IT-TOF-MS: liquid chromatography–ion trap–time of flight–mass spectrometry; HPLC: high-performance liquid chromatography; ABTS^+^: the 2-azino-bis(3-ethylbenzothiazoline-6-sulfonic acid diammonium salt; DPPH: 1,1- diphenyl-2-picrylhydrazyl; FRAP: Ferric reducing antioxidant power; DNS: 3,5- dinitrosalicylic acid; PCA: principal component analysis: PLS-DA: partial least squares discriminant analysis; OPLS-DA: orthogonal partial least squares discriminant analysis; PPO: polyphenol oxidase; POD: peroxidase.
