# Supplementary material for: Evaluate the Effects of Different Processing Methods on Red Dragon Fruit (Hylocereus species) Juice from the Perspective of Physicochemical Properties and Metabolic Profiles
Source: Foods. 2025 Feb 26;14(5):793. doi: 10.3390/foods14050793 (PMC11899162; doi:10.3390/foods14050793)
Supplement: Supplementary file 1 [file foods-14-00793-s001.zip › foods-3479577-supplementary.pdf]

Evaluate the effects of different processing methods on red dragon fruit (*Hylocereus species*) juice from the perspective of physicochemical properties and metabolic profiles

Shuai Chen [1], Yansong Li [1], Jiamu Kang [1,2], Congyan Su [1], Yuyi Liu [1], Yanfei Cheng [1], Zexin Wang [1], Shuxian Li [1], Congfa Li [1,2,3,*]

[1] School of Food Science and Engineering, Hainan University, Haikou 570228, China
17674726015@163.com (S.C.); 18834167777@163.com (Y.L.); kangjiamu@hainanu.edu.cn (J.K.); 21110832000007@hainanu.edu.cn (C.S.); 22220951350082@hainanu.edu.cn (Y.L.); chengyanfei1620@163.com (Y.C.); 18313570568@163.com (Z.W.); 22110832000009@hainanu.edu.cn (S.L.)

[2] Key Laboratory of Food Nutrition and Functional Food of Hainan Province, Haikou 570228, China

[3] Key Laboratory of Tropical Agricultural Products Processing Technology of Haikou, Haikou 570228, China

* Correspondence: congfa@hainanu.edu.cn; Tel.: +86-898-66193026

Table S1. Concentrations of 157 VOCs

No .	Compounds name	CAS	RT	RI	Concentration（μg/mL）			
					RAJ	CP	US	PT
1	Benzyl alcohol	100-51-6	31.6	1871	0.066±0.007a	0.014±0.005c	0.035±0.006b	ND
2	(2R,3R)-(-)-2,3-Butanediol	24347-58-8	22.236	1576	0.301±0.068b	0.753±0.106a	0.088±0.013c	ND
3	Phenylethyl Alcohol	60-12-8	32.59	1905	0.399±0.010a	0.182±0.016b	0.102±0.013c	0.034±0.011d
4	Geranyllinalool	1113-21-9	44.518	2303	0.205±0.021a	ND	ND	ND
5	1-Hexadecanol	36653-82-4	39.86	2147	ND	0.153±0.078a	0.048±0.007a	0.256±0.329a
6	(3S)-3-Methyl-1-pentanol	42072-39-9	39.867	2148	ND	0.107±0.056b	ND	0.373±0.015a
7	3,4-dimethylbenzyl alcohol	6966-10-5	38.148	2090	ND	0.037±0.002a	ND	ND
8	1-Nonanol	143-08-8	24.882	1656	ND	0.047±0.004b	0.019±0.006c	0.063±0.008a
9	(Z)-2-nonen-1-ol	41453-56-9	26.615	1709	ND	0.046±0.006a	ND	0.027±0.008b
10	Costol	515-20-8	29.094	1788	ND	0.068±0.006a	ND	ND
11	1-Dodecanol	112-53-8	39.863	2147	ND	ND	0.054±0.035a	ND
12	1-Undecanol	112-42-5	13.616	1328	ND	ND	0.047±0.007a	ND
13	(5-Ethylcyclopent-1-enyl)methanol	36431-59-1	25.043	1661	0.021±0.003a	ND	ND	ND
14	β,2,3-trimethyl-Cyclopentaneethanol	21721-18-6	45.264	2328	ND	ND	ND	0.015±0.001a
	Alcohols							
15	2,4-Di-t-butylphenol	96-76-4	43.452	2267	ND	0.068±0.009a	ND	ND
16	2-METHOXYFURAN	25414-22-6	7.343	1133	0.242±0.009a	0.19±0.040ab	0.116±0.012c	0.136±0.010b c
17	2-Amylfuran	3777-69-3	9.568	1208	0.071±0.008a	0.086±0.005a	0.085±0.020a	0.038±0.007b
18	Coumaran	496-16-2	45.389	2332	0.090±0.016b	0.045±0.019b	0.010±0.006b	5.830±0.227a
	Furans							
19	P-Xylene	106-42-3	7.287	1131	0.144±0.019b	0.062±0.002c	0.241±0.030a	0.064±0.004c
20	Dimethyl ether	115-10-6	3.589	922	13.375±1.451	8.10ND.643	4.164±1.122c	11.197±1.311

					a	b		a
21	4-Ethyltoluene	622-96-8	9.362	1202	0.034±0.002b	0.062±0.006b	0.052±0.006b	0.726±0.065a
22	1,2,4-TRIMETHYL BENZENE	95-63-6	11.351	1262	3.045±0.987a	2.870±0.227a	1.869±0.390a	ND
23	2-methyl-1,3-Dioxolane	497-26-7	0.15	654	0.004±0.001a	ND	ND	ND
24	Decanoic anhydride	2082-76-0	29.984	1817	ND	ND	0.044±0.002a	ND
25	Alloaromadendrene oxide-(1)	1000156-12-8	29.082	1788	ND	ND	0.068±0.010a	ND
26	(-)-Isolongifolol, methyl ether	1000333-80-8	32.487	1901	ND	ND	ND	0.124±0.023a
	Others							
27	2,4-dimethylbenzaldehyde	15764-16-6	29.501	1801	0.645±0.076a	0.400±0.050b	0.394±0.030b	NDc
28	Apricolin	104-61-0	35.858	2014	0.316±0.013a	ND	ND	ND
29	undecenal	2463-77-6	27.661	1743	0.081±0.003a	ND	ND	ND
30	(2E,4E)-Deca-2,4-dienal	25152-84-5	35.254	1993	0.076±0.005a	ND	ND	ND
31	(2E)-2-Decenal	3913-81-3	24.174	1634	0.056±0.003a	ND	ND	ND
32	pentadecanal	2765-11-9	32.964	1917	ND	0.016±0.001c	0.034±0.003b	0.048±0.005a
33	(Z)-13-Octadecenal	58594-45-9	38.373	2098	ND	0.062±0.005a	0.052±0.002a	0.206±0.264a
34	Benzaldehyde	100-52-7	35.426	1999	ND	ND	ND	0.017±0.002a
35	(2E)-2-Nonenal	18829-56-6	36.877	2048	ND	ND	ND	0.411±0.041a
36	2,5-Dimethylbenzaldehyde	5779-94-2	14.44	1352	ND	ND	ND	0.126±0.004a
37	(2E,4E)-2,4-Nonadienal	5910-87-2	21.718	1561	ND	ND	ND	0.019±0.006a
38	5-Ethylcyclopent-1-enecarboxaldehyde	36431-60-4	24.892	1656	ND	ND	ND	0.024±0.005a
39	(E,Z)-2,6-Nonadienal	557-48-2	22.328	1578	ND	ND	ND	0.035±0.006a
	Aldehydes							
40	Octanoic acid	124-07-2	36.869	2047	1.455±0.064a	1.146±0.114b	0.737±0.095c	0.860±0.0240
								c
41	1-Hexanoic acid	142-62-1	30.765	1844	1.176±0.104a	0.859±0.042b	0.481±0.025c	0.266±0.055d
42	Lauric acid	143-07-7	47.536	2404	0.163±0.025a	0.073±0.003b	0.076±0.003b	0.026±0.003c

43	Decanoic acid	334-48-5	42.432	2233	0.125±0.021b	0.179±0.029a	0.047±0.008c	0.064±0.008c
44	Palmitoleic acid	373-49-9	41.103	2189	2.848±0.310a	3.648±0.574a	1.466±0.064b	1.656±0.107b
45	Myristic acid	544-63-8	52.247	2561	0.106±0.013bc	0.347±0.009a	0.081±0.005c	0.123±0.016b
46	Palmitic acid	57-10-3	57.247	2728	0.211±0.008c	0.557±0.038a	0.133±0.018d	0.362±0.033b
47	Acetic acid	64-19-7	25.724	1681	5.056±0.724b	14.257±1.139a	5.190±0.900b	1.888±0.128c
48	3-Methylvaleric Acid	105-43-1	25.347	1670	0.21±0.01a	ND	ND	ND
49	Oxalic acid	144-62-7	3.811	939	0.362±0.059a	ND	ND	ND
50	1,2-Benzenedicarboxylic acid	84-74-2	52.167	2558	ND	0.025±0.004a	ND	ND
51	Nonanoic acid	112-05-0	39.711	2142	ND	ND	0.006±0.001a	ND
52	Malonic acid	141-82-2	2.536	840	ND	ND	ND	ND
	Acids							
53	longicyclene	1137-12-8	18.79	1475	0.109±0.012a	0.073±0.012b	0.079±0.007b	0.074±0.007b
54	(+/-)-13-methyl-tridecanolide	27198-63-6	35.739	2010	1.001±0.105b	1.373±0.204a	0.558±0.033c	0.672±0.082c
55	3-Methyl-5-Propylnonane	31081-18-2	22.865	1594	0.039±0.003b	0.087±0.007a	ND	ND
56	(+)-Longifolene	475-20-7	21.296	1548	2.089±0.280a	1.482±0.097b	1.469±0.092b	1.312±0.221b
57	Tridecane	629-50-5	11.499	1266	3.551±0.307a	2.158±0.291b	2.599±0.232b	2.311±0.271b
58	Dodecane	112-40-3	8.233	1164	0.021±0.001a	ND	0.020±0.005a	0.008±0.001b
59	2-methyl-3-Octyne	55402-15-8	16.396	1407	0.016±0.005a	ND	0.020±0.003a	ND
60	α-longipinene	5989-08-2	17.714	1445	0.459±0.052a	ND	ND	0.338±0.031b
61	pentadecane	629-62-9	19.285	1489	0.161±0.026a	ND	ND	0.044±0.004b
62	N-HEPTADECANE	629-78-7	26.25	1698	0.068±0.012a	ND	ND	ND
63	1,2-Epoxyhexadecane	7320-37-8	32.927	1916	0.025±0.001a	ND	ND	ND
64	3,3-Dimethylhexane	563-16-6	15.879	1393	ND	0.012±0.001a	ND	ND

65	N-hexane	110-54-3	7.153	1127	ND	ND	ND	0.042±0.018a
66	(E,E)-7,11,15-Trimethyl-3-methylene-hexadeca-1,6,10,14-tetraene	70901-63-2	41.999	2219	ND	ND	ND	0.035±0.004a
67	α-Terpinene	99-86-5	18.57	1469	ND	ND	ND	0.037±0.001a
68	6-Tridecene	24949-38-0	13.125	1314	ND	ND	ND	0.042±0.003a
	Hydrocarbon							
69	4-Methyl-2-pentanone	108-10-1	4.612	1001	8.62±1.162a	2.106±0.081b	1.715±0.152b	1.059±0.198b
70	2,6-Dimethyl-4-heptanone	108-83-8	8.103	1159	0.577±0.058a	0.463±0.035a	0.488±0.091a	0.288±0.019b
71	4,6-Dimethyl-2-Heptanone	19549-80-5	10.306	1231	0.159±0.005a	0.127±0.012b	0.072±0.005c	0.058±0.003c
72	Acetoin	513-86-0	12.859	1307	10.47±0.967a	2.233±0.363b	3.757±0.605b	0.062±0.010c
73	2-Heptanone	110-43-0	8.555	1175	0.142±0.029a	ND	ND	ND
74	Methyl nonyl ketone	112-12-9	22.696	1589	0.019±0.003a	ND	ND	ND
75	oxacyclododecan-2-one	1725-03-7	38.347	2097	ND	ND	0.03±0.005a	ND
76	4,6-Dimethyl-5-hepten-2-one	31162-48-8	7.861	1151	ND	ND	0.008±0.001a	ND
77	5-hydroxy-2,7-dimethyl-4-Octanone	6838-51-3	26.19	1696	ND	ND	ND	0.048±0.002a
78	2,3-Butanedione	431-03-8	4.171	967	ND	ND	ND	0.286±0.031a
	Ketone							
79	Ethyl phenylacetate	101-97-3	28.803	1779	0.058±0a	0.029±0.001b	0.024±0.003c	0.033±0.001b
80	Ethyl heptanoate	106-30-9	13.318	1320	0.134±0.02a	0.085±0.002b	0.054±0.002c	0.026±0.003d
81	Ethyl laurate	106-33-2	30.601	1838	1.879±0.148a	0.902±0.064b	0.877±0.654b	1.235±0.047ab
82	1,3-dimethylbutyl acetate	108-84-9	6.504	1104	1.026±0.056b	1.436±0.094a	0.448±0.023c	0.106±0.006d
83	Ethyl caprate	110-38-3	24.048	1630	1.064±0.021a	0.925±0.093ab	0.653±0.037c	0.773±0.066bc
84	Ethyl nonanoate	123-29-5	20.559	1527	0.139±0.004a	0.136±0.032a	0.107±0.023a	ND
85	Ethyl hexanoate	123-66-0	9.877	1218	0.829±0.097a	0.475±0.020b	0.384±0.021b	0.430±0.021b

86	Ethyl tetradecanoate	124-06-1	36.566	2037	2.612±0.154a	2.791±0.505a	1.727±0.309b	1.800±0.095b
87	Ethyl acetate	141-78-6	3.115	885	0.346±0.019a	0.152±0.040b	0.202±0.007b	0.025±0.006c
88	Methyl 2-hydroxy-4-methylbenzoate	4670-56-8	33.955	1950	0.288±0.032a	0.252±0.030a	0.135±0.01b	0.054±0.007c
89	Ethyl palmitate	628-97-7	41.996	2219	0.661±0.037b	0.645±0.039b	0.269±0.024c	1.732±0.117a
90	Ethyl caprylate	106-32-1	16.974	1424	4.650±0.390a	NDd	2.273±0.265c	3.138±0.207b
91	Formic acid	112-32-3	21.471	1553	0.037±0.006a	ND	ND	ND
92	Linalyl formate	115-99-1	21.131	1543	0.073±0.006a	ND	ND	ND
93	(3Z)-3-Hexen-1-yl formate	33467-73-1	15.631	1385	0.013±0.002a	ND	ND	ND
94	13,16-Octadecadiynoic acid methyl ester	56846-98-1	29.293	1795	0.093±0.011a	ND	ND	ND
95	Ethyl undecanoate	627-90-7	27.372	1733	0.032±0.009a	ND	ND	ND
96	2-Ethylbutyl hexanoate	91933-26-5	16.585	1413	0.441±0.033a	ND	0.271±0.006b	0.166±0.014c
97	Methyl octylate	111-11-5	15.583	1384	ND	0.015±0.004b	ND	0.038±0.015a
98	lavandulyl acetate	25905-14-0	29.303	1795	ND	0.136±0.040a	ND	ND
99	Heptyl Acetate	112-06-1	15.035	1369	ND	0.008±0.001a	ND	ND
100	Ethyl pentadecanoate	41114-00-5	39.341	2130	ND	0.039±0.005a	ND	0.045±0.013a
101	(E,E)-farnesyl acetate	4128-17-0	32.42	1899	ND	ND	0.046±0.003a	ND
102	Ethyl undecylenate	692-86-4	32.581	1904	ND	ND	ND	0.041±0.006a
103	Lauric acid, isohexyl ester	1000438-93-1	35.882	2014	ND	ND	0.073±0.010a	ND
104	Octanoic acid, 3-methylbutyl ester	2035-99-6	24.714	1651	ND	ND	ND	0.016±0.004a
	Esters							

Note: Letters indicated statistically significant differences, ND means not detected

Table S2: pH and TS of different treatments

Treatments	pH	TSS
RAJ	4.77±0.03[b]	12.66±0.01[a]
PT	4.83±0.01[a]	12.53±0.01[b]
US	4.76±0.01[b]	12.73±0.10[a]
CP	4.79±0.02[b]	12.73±0.10[a]
